# “Guilty until proven innocent”: the contested use of maternal mortality indicators in global health

**DOI:** 10.1080/09581596.2016.1259459

**Published:** 2016-12-20

**Authors:** Katerini T. Storeng, Dominique P. Béhague

**Affiliations:** ^a^ Centre for Development and the Environment, University of Oslo, Oslo, Norway; ^b^ The Center for Medicine, Health and Society, Vanderbilt University, Nashville, TN, USA; ^c^ Department of Global Health and Social Medicine, Kings College London, London, UK; ^d^ London School of Hygiene & Tropical Medicine, London, UK

**Keywords:** Global health, anthropology, indicators, maternal mortality, maternal health, ethnography

## Abstract

The MMR – maternal mortality ratio – has risen from obscurity to become a major global health indicator, even appearing as an indicator of progress towards the global Sustainable Development Goals. This has happened despite intractable challenges relating to the measurement of maternal mortality. Even after three decades of measurement innovation, maternal mortality data are widely presumed to be of poor quality, or, as one leading measurement expert has put it, ‘guilty until proven innocent’. This paper explores how and why leading epidemiologists, demographers and statisticians have devoted the better part of the last three decades to producing ever more sophisticated and expensive surveys and mathematical models of globally comparable MMR estimates. The development of better metrics is publicly justified by the need to know which interventions save lives and at what cost. We show, however, that measurement experts’ work has also been driven by the need to secure political priority for safe motherhood and by donors’ need to justify and monitor the results of investment flows. We explore the many effects and consequences of this measurement work, including the eclipsing of attention to strengthening much-needed national health information systems. We analyse this measurement work in relation to broader political and economic changes affecting the global health field, not least the incursion of neoliberal, business-oriented donors such as the World Bank and the Bill and Melinda Gates Foundation whose institutional structures have introduced new forms of administrative oversight and accountability that depend on indicators.

## Introduction

The maternal mortality ratio (MMR) has risen from obscurity to become a major global health indicator. A measure of the number of women dying of pregnancy-related causes per 100,000 live births, the MMR indicates the population-level risk of maternal death. It is also widely regarded as an indicator of health system functioning because improving maternal survival depends on a health system that is able to refer women to and deliver high-skilled emergency care. More recently, the MMR has achieved prominence as an indicator of progress towards global development goals, including the Millennium Development Goals (MDGs) and the Sustainable Development Goals that have replaced them (Fukuda-Parr, Yamin, & Greenstein, 2014). But while MMR estimates have helped to secure a place for maternal survival on the global policy agenda (Smith & Rodriguez, 2015), the maternal health field has also struggled to contend with persistent measurement challenges. According to a prominent UK-based epidemiologist we interviewed, measurement problems related to the MMR have been a ‘thorn in the side of the MDG agenda’ and even after decades of measurement innovation, one expert told us that maternal mortality data are widely presumed ‘guilty until proven innocent’ (Storeng & Béhague, 2014).

This paper aims to examine the development of the MMR and the various contestations and practices that professionals have engaged in as they chart out what the MMR actually reveals, obscures and does. As responsibility for the production and analysis of data has, at least partially, shifted from national Ministries of Health to ‘global’ institutions and scientists (Harper, 2006; Rees, 2014), it becomes important to examine the roles, perspectives and practices of such scientists and their professional communities. In ‘studying up’ (Nader, 1972), we explore how demographers, statisticians and epidemiologists, active in international efforts to improve maternal health in low- and middle-income countries, are charged with making the numbers appear robust, and indeed ‘innocent’.

We now live in an era in which metrics have been embraced as central to the solution of intractable global health problems (Adams, 2016b). It is thus not surprising that the international Task Force on Child Health and Maternal Health (2005, p. 78) more than a decade ago claimed that ‘the analysis of maternal mortality, the range of possible solutions, and the need for priority setting begins with numbers’. Advocates of this position often argue that statistical indicators, rankings and monitoring are central to the functioning of the various public–private partnerships that have formed in the past 15 years, including the Partnership for Maternal, Newborn and Child Health. Measurement underlies ‘greater commitments to transparency and accountability’ and allows ‘better understanding of what works: how many lives have been saved and at what cost’ (Boerma & Abou-Zahr, 2007, p. 718).

We argue that an equally important driver of the emphasis on maternal mortality measurement is experts’ perceived need to respond to donors’ demand for data to justify and monitor the results of investment flows, and to produce data that can help secure – and sustain – global-level political priority for safe motherhood in an increasingly competitive policy space (Storeng & Béhague, 2014). While being cognisant of the dangers of invoking the term neoliberalism in public health critique (Bell & Green, 2016), it is clear that measurement experts’ work is increasingly linked to what Adams (2016a, p. 45) has recently called the ‘micropractices of neoliberalism’. These practices are ‘*not necessarily* designed with neoliberal outcomes in mind’, but they ‘work seamlessly with the political aspirations of neoliberal reforms’ (Adams 2016a, p. 45).

In our analysis, we use the term ‘neoliberal’ to refer quite specifically to the assumption that health care priorities setting should be shaped primarily by cost-effectiveness logics and business management models. This assumption has become mainstream in the past two decades as part of the transition from international, intergovernmental health collaboration to ‘global health’, characterised by market-driven approaches and public–private partnerships for health (Birn, Nervi, & Siqueira, 2016). These developments have been enabled in large part by the rise of neoliberal and business-oriented institutions like the World Bank and the Bill & Melinda Gates Foundation as key authorities within global health governance (Brown, Cueto, & Fee, 2006; Fee, Cueto, & Brown, 2008). These actors’ institutional practices have introduced new forms of administrative oversight, audit and accountability, a clear manifestation of the incursion of corporate thinking and culture into broader social spheres (Merry, 2011). As Erikson (2015) has shown, today’s dominant global health actors tie market interests to health outcomes in ways that depend on numerical indicators. The growing power of private foundations in global health governance has also supported the proliferation of competing ‘advocacy coalitions’ and issue-specific global health networks (Shiffman et al., 2016), whose success is increasingly defined by their ability to reach concrete goals through management-style performance accountability measures that depend on health data*.* This form of accountability provides a stark contrast to the broader social justice goals that defined success in earlier stages of the history of international health (Birn, 2009).

We aim to show how these assumptions have influenced the heart of scientific practice, encouraging measurement scientists to accommodate donors’ need for indicators to monitor investment flows, ensure financial accountability and justify their claims that they are saving lives. Although measurement experts are motivated by broader concerns to advance science and to strengthen national and local health systems, such aims sometimes become subservient to donors’ and international agencies’ needs for ‘global’ accountability data. Most notably, the imperative to respond to donors’ data demands has encouraged the development of ever more sophisticated and expensive surveys and mathematical models to provide globally comparable estimates of maternal mortality that serve to make messy numbers appear robust, but that eclipse the building up of much needed national health information systems.

What tensions do measurement experts themselves experience over the use of the MMR as the key indicator by which progress towards improved maternal health – and towards various politically important targets – is assessed? In what follows, we show that despite the consensus that making MMR data credible is critical for advocacy, measurement experts sometimes downplay the uncertainties of the MMR out of fear that these uncertainties will lead donors to invest elsewhere. Yet privately, they criticise, and sometimes resist, the ways the MMR is used and the donor demands for data that make the MMR so central relative to both other numerical indicators and broader forms of evidence. We examine how measurement experts become involved in controversies over the numbers that are not just technical in nature, but that reveal deep-seated power struggles and ideological differences, such as those between international public agencies like the WHO and private donors like the Gates Foundation. We highlight the ways that different donors impact on how – and what kinds of – data hold sway. At stake, then, is not just the meaning of the numbers, but how to make them meaningful (as evidence of success) to the people who matter. Ironically, the people who matter are not the women who benefit from maternal health interventions, but the donors who fund them.

## Methodology

Our analysis forms part of a broader ethnographic project on the international safe motherhood movement and its responses to the rise of evidence-based policy-making (Béhague & Storeng, 2008; Béhague & Storeng, 2013; Storeng & Béhague, 2014). To understand the making of the MMR, we draw on participant observation over the past decade within research communities, primarily in the UK, and at policy events. Our ethnographic access was facilitated by our involvement in an interdisciplinary Research Programme Consortium on Maternal and Newborn Health funded by the UK Department for International Development and Immpact, a major research initiative on maternal mortality measurement. We also conducted in-depth interviews between 2004 and 2009 with 72 informants from multilateral agencies, academic institutions, professional bodies and international non-governmental organisations, primarily at the global level. This paper is based largely on interviews with the 23 informants who were international academic researchers. This epistemic community consisted primarily of measurement experts (epidemiologists, statisticians and demographers, many with a clinical background) based at elite global health research institutes and universities in Europe and North America. To understand the evolving debates about maternal mortality measurement, we also draw on historical and contemporary policy documents and scientific literature and oral histories collected during in-depth interviews.

## Measurement bottlenecks

‘Sound estimates based on new data … are the foundation of our current understanding and concern,’ said WHO’s Director General Halfdan Mahler when addressing the Nairobi conference that launched the international Safe Motherhood Initiative (SMI) in 1987 (Mahler, cited in AbouZahr, 2003). Mahler was referring to the first global maternal mortality estimates published by the WHO in 1986, showing that around 500,000 women were dying annually from causes relating to pregnancy and childbirth, 99% of them in poor countries (WHO, 1986).

In reality, however, these estimates were not ‘sound’ because most low- and middle-income countries lacked reliable systems for registering births and deaths, while many others could not produce statistics on mortality by age, sex and cause of death, which are considered basic indicators for understanding a country’s public health profile. The dearth of data in the poorer countries was in stark contrast to high-income countries. In such countries, civil registration systems, parish registers and recording of maternal deaths in maternity services had enabled analyses of trends in maternal mortality since the nineteenth century (Loudon, 1992), and have been considered essential to these countries’ ability to dramatically reduce maternal mortality (Béhague & Storeng, 2013). In the absence of empirical measurement in poor countries, the WHO used approximation methods to create the estimates, for instance adjusting the deaths to women of reproductive age by the proportion estimated to be due to maternal causes – often assumed to be 25–33% (AbouZahr, 1998).

From the late 1980s, academics from leading public health schools such as the London School of Hygiene and Tropical Medicine in the UK and Johns Hopkins and Columbia University in the US collaborated with WHO statisticians to develop new approaches to estimating maternal mortality. These were intended as interim solutions until countries developed functioning vital registration systems capable of capturing cause-of-death data. In line with wider developments in the production of health and vital statistics, such as the USAID-funded Demographic and Health Surveys, epidemiologists, demographers and statisticians at these British and American universities worked on methods to document maternal deaths as they occurred in the whole population, including among those women who delivered at home. The best known of these, developed by a team led by British epidemiologist Wendy Graham, was known as the sisterhood method, an indirect method for estimating maternal mortality based on asking adults during a census or survey about deaths during pregnancy and childbirth among their adult sisters (Graham, Brass, & Snow, 1989).

These methodological innovations were driven by a belief in what Graham and her colleague Oona Campbell referred to as a ‘measurement trap’ (Graham & Campbell, 1992). The measurement trap stipulated that lack of data to establish the levels and trends of specific maternal health outcomes, to identify the characteristics and determinants of these outcomes, and to monitor and evaluate the effectiveness of programmes, was leading to neglect of maternal health in research and programmes in a self-reinforcing cycle. This idea of the ‘measurement trap’ became a key trope in the SMI’s historical self-narrative. As one of the initiative’s founding members explained:

It was always measurement. That was always the focus. It was always the belief that part of the problem was in measurement bottlenecks and generally feeling that by improving indicators and measurement techniques that we would help to address part of the problem. So the problem was neglected because there wasn’t enough information, there wasn’t enough information so it was all a vicious circle.

However, experts explained that measurement challenges specific to maternal mortality in low-income countries had hampered attempts to break this ‘vicious cycle’. The classification of maternal mortality, a widespread tendency to underreport maternal causes in cause-of-death surveys and poor quality of available data sources had made it difficult to produce accurate numbers (Campbell & Graham, 1990). Moreover, collecting maternal mortality data through surveys was both labour-intensive and expensive, because maternal deaths are relatively infrequent at a population level, thus requiring large sample sizes to achieve statistical precision (Merdad, Hill, & Graham, 2013). In the Safe Motherhood Initiative’s early years, experts debated whether the cost was justified. As Graham has recalled, many came to feel that measuring maternal mortality using surveys ‘was pretty much impossible and definitely not an efficient use of scarce resources’ (Graham, 2002:701). During this period, many leading epidemiologists therefore also worked on developing indicators that could serve as proxies for maternal mortality. For example, many worked on developing indicators of severe obstetric morbidity, since such morbidity had been shown to occur much more frequently than maternal deaths and was therefore easier to measure. Others developed process indicators to assess the provision of and women’s access to emergency obstetric care services, necessary for treating obstetric complications such as haemorrhage, sepsis and obstructed labour, which had been identified as the main direct causes of maternal deaths (Maine, 1991).

Although there was a strong focus on maternal mortality in these early measurement efforts, many attempted to keep multiple agendas on the table. The same epidemiologists who led the maternal mortality measurement agenda also argued that the focus on mortality was too narrow. Graham and Campbell (1992, p. 967), for example, claimed that efforts to dismantle the measurement trap ‘revealed a weak conceptual framework to lie at the very centre’. ‘Maternal health,’ they noted, ‘has tended to be conceptualised as a discrete, negative state, characterised by physical rather than social or mental manifestations, and by a narrow time-perspective focusing on pregnancy, delivery and the puerperium’ (Graham & Campbell, 1992, p. 967). Given these limitations, they called for a broader perspective and broader indicators of women’s reproductive health beyond pregnancy.

## From measurement for local government to measurement for global accountability

Despite such calls for a broader approach to maternal health indicators, in the second decade of the SMI, measurement work was increasingly directed more narrowly towards supplying a growing donor-driven demand for ‘global’ maternal mortality estimates. This reflected the growing preoccupation with financial accountability and ensuring cost-effectiveness during this period. According to informants who worked within the field at this time, UN agencies were committed to publishing new global maternal mortality estimates every five years, both because governments wanted estimates to justify their claims for donor support and because international donors required mortality data as evidence of the impact their support was having on saving lives.

The move towards regular monitoring was initiated largely by the World Bank, which, in the early 1990s, staged what historians have depicted as an aggressive assault on the WHO’s authority within international health (Fee et al., 2008). Its *Investing in Health* report, published in 1993, mainstreamed the idea that governments and donors should prioritise selective cost-effective health interventions targeting high burden health problems, leaving the rest to the private sector (World Bank, 1993). Cost-effectiveness was to be assessed using the DALY (disability adjusted life year), a new metric created to quantify the overall disease burden from specific health conditions on a global scale, expressed in number of years lost due to ill health (Murray & Acharya, 1997). The use of cost-effectiveness analysis to guide priority setting was buttressed by the expansion of evidence-based policy-making to countries receiving aid, notably the use of randomisation in controlled trials to evaluate clinical effectiveness (Dobrow, Goel, & Upshur, 2004; Lambert, 2006).

The rise of new private donors around the turn of the Millennium, most notably the Gates Foundation, further entrenched the idea that investments in health should provide ‘value for money’ (Birn, 2014; McCoy & McGoey, 2011). From its establishment in 1999, the Gates Foundation quickly became a dominant actor in global health, influencing the behaviour of other donors too. It oversaw the establishment of major global public–private partnerships for health, notably GAVI, the Vaccine Alliance and the Global Fund, oriented towards the achievement of global goals for child survival and HIV/Aids, TB and Malaria, respectively (Brown et al., 2006). Within a few years, nearly 100 public–private partnerships or global health initiatives had formed, competing with each other for donor resources, while donor-dependent governments competed for grants and other investments from these new actors (Buse & Harmer, 2007).

Maternal health experts we spoke with noted how swiftly both public and private donors adopted an emphasis on ‘value for money’ as the ultimate measure of an intervention’s worth. As one USAID-funded NGO representative said in an interview in Washington, D.C. in 2005, ‘donors never wanted indicators and then they wanted results and everybody started asking “what are you using your money for”... they want to see exactly “how many lives my $500,000 has saved.” How many maternal lives were saved?’ This preoccupation also reflected intense emphasis on monitoring progress towards the MDGs, itself justified in terms of the need for accountability and transparency.

## The measurement trap and the competition for political priority

The maternal health field – from policy-makers, to advocacy specialists, to academic researchers – struggled to respond to the exigencies of this hypercompetitive, results-oriented environment. Despite the methodological innovations in maternal mortality estimation of the 1990s, by 2004, the WHO (2004) noted that ‘problems of underreporting and misclassification are endemic to all (these) methods’. The UN’s global estimates continued to be based on regression models or approximation methods rather than ‘real’ measures, because most countries with high levels of maternal mortality did not have a reliable system of civil registration (WHO, 2004). Population-based surveys using techniques such as the sisterhood method were the dominant data source, but produced estimates that were imprecise (subject to wide margins of uncertainty) as a result of sample size issues, making it difficult to provide firm statements about global maternal mortality trends (WHO, UNICEF, & UNFPA, 2004, p. 4). With global estimates stubbornly stagnant at around 500,000 deaths annually, measurement experts like WHO’s Carla AbouZahr (2001, p. 390) noted that ‘it became increasingly difficult to keep maternal health in the public eye when there was nothing new to report’. Even though the inclusion of maternal health among the Millennium Development Goals generated political commitment, many of our informants worried about how to sustain interest given the difficulties with monitoring progress. As we were told more than once: ‘a goal that cannot be measured cannot be monitored or met’.

Given the practical challenges of producing annual maternal mortality estimates, a new indicator, ‘skilled birth attendance’ – deliveries attended by a health worker with midwifery skills – was introduced as a proxy indicator for measuring global progress towards the MDGs. But there were measurement challenges associated with this indicator too, not least related to the fact that the category of health worker classified as ‘skilled’ varies widely across settings. Moreover, key actors questioned the assumption that institutionalising deliveries in settings with high levels of home births would reduce mortality, without concomitant changes to the quality of care and acceptability of services offered (see Austveg, 2011; Spangler, 2012). Maternal health advocates we spoke with, and measurement experts too, were candid that demonstrating change in the skilled birth attendance indicator would not have the same political power as demonstrating a reduction in the MMR (see also Austveg, 2011).

The lack of ‘gold standard’ RCT-evidence of the impact of proposed interventions on maternal mortality compounded concerns about how to monitor progress towards global goals (Béhague & Storeng, 2008). As our informants explained, producing such evidence is prohibitively expensive and logistically challenging; because maternal mortality is relatively infrequent on a population basis, huge sample sizes are needed to achieve statistical precision. Nevertheless, they explained, donors use the lack of such evidence of impact of proposed interventions to place their money elsewhere.

## Now or never

With researchers increasingly cognisant of the political importance of mortality data, they quickly recast improving maternal mortality measurement as a global priority. Wendy Graham (2002, p. 703) even said better measurement was a case of ‘now or never’

We must stop saying this [maternal mortality measurement] cannot be tackled and acknowledge the damage caused so far. We must recognise the risks of continuing to neglect the data needed by poor countries to inform their allocation of scarce resources, and find the funds, the tools, and the opportunities to meet these needs. We must build a sustainable evaluation capacity at the country level and a greater demand for reliable measurement of maternal mortality…

The irony that this appeal came from Graham, who, a decade earlier had argued vehemently that the MMR was too narrow as an indicator of maternal health, was not lost on our informants. As one of Graham’s close colleagues, explained, ‘[We] had co-authored a paper saying that you shouldn’t be measuring maternal mortality … And then Wendy came back … going for the argument that people really want maternal mortality.’ But, as a senior medical doctor and SMI founder elaborated, this renewed emphasis on mortality measurement was a direct response to the Gates Foundation and other donors who were ‘reluctant to put money into safe motherhood and sort of asked the question, “do you in fact not know what to do and would it be worthwhile investing in helping you to find out what to do?”’ Graham had argued, he recalled, that ‘you can only identify what are the effective policies if you can first of all measure maternal mortality…first of all we need years of improving measuring and then we will test policies.’ She persuaded the Gates Foundation and the British and American international development agencies to fund a seven-year research programme called Immpact, the Initiative for Maternal Mortality Programme Assessment. Immpact promised to develop new measurement methods ‘in order to strengthen capacity for evidence-based decision-making and rigorous evaluation’ and ‘to determine and evaluate cost-effective interventions and strategies for improvement,’ though struggled to deliver on this promise given the measurement challenges discussed above (University of Aberdeen, 2016).

During this period, major donors also funded measurement experts to contribute to new global accountability mechanisms, notably the Countdown to 2015 for Maternal, Newborn and Child Survival, initiated in 2003. This initiative tracked progress towards MDG 4 and 5 on child survival and maternal health in 75 ‘priority’ countries. According to some of those who participated, it was premised on the idea that such monitoring would incentivise governments and donors to prioritise maternal mortality reduction. Many measurement experts were also involved in UN-led efforts to improve the mathematical models and approximation methods used for producing global maternal mortality estimates. Through involvement in Immpact, the Countdown and similar initiatives, leading measurement experts thus became increasingly involved in supplying the methods and data needed to ensure accountability and value for money of donor investments.

Academics’ willingness to engage in such practices reflects that many construed the problems with the maternal mortality indicators as central drivers of their continued struggle to make maternal health a global priority. One informant articulated a common view when she said, ‘a major factor in terms of the difficulties with achieving progress, has been the difficulty of measuring it … if you can’t measure it, you don’t do it’. This view became mainstream following the 2007 publication of political scientist Jeremy Shiffman’s (2007, p. 1370) case study of the generation of political priority for safe motherhood. Shiffman argued that attracting political support for maternal mortality had been difficult precisely because of the technical difficulties with measuring maternal mortality and documenting progress, compounded by the fact that maternal deaths were not as common as those caused by other high-burden problems like HIV/AIDS and malaria. His take-home message was that improved maternal mortality measurement would be key to generating resources and political attention.

## Science and advocacy

With health indicators poised as key instruments of competition and governance, measurement experts found themselves under new pressure to participate in more active ways in global-level advocacy and policy processes. Often, they were called upon to provide scientific legitimacy to the numbers, where their impulse to emphasise the uncertainty associated with maternal mortality estimates came into conflict with the pressure to provide unequivocal policy-relevant conclusions. An American epidemiologist conceded that the ‘quick-winnism of the taskmasters … has pushed a lot of people to simplify their message’. Although the measurement limitations specific to maternal mortality are no secret within the global health field, measurement experts feared that donors would seize on statistical uncertainty as an excuse to divert resources to other global health investments that can more conclusively be shown to offer ‘value of money’. Debating scientific uncertainty or alternative interpretations of existing data could therefore, some felt, undermine donor commitment to maternal health. As one expert put it: ‘They say they won’t do anything where there isn’t agreement’.

The production of the Lancet series on maternal health, published in 2006, aptly illustrates academics’ increasing confrontation with professionalised global health advocacy. The series intended to present the state-of-the-art on maternal health, along with an evidence-based justification for prioritisation at a time when the MDG on maternal health was perceived to be lagging. A leading epidemiologist at a British public health school recalled her discomfort upon realising, during an editorial meeting she participated in, that she was expected to draw much firmer conclusions about global trends than she felt comfortable doing on the basis of the existing data:

They wanted us to say ‘look how big the problem is. Countries make no progress, yet we know what works and what needs to be done’. That’s what people wanted to hear. They called it ‘evidence-based advocacy’… My feeling is that it’s slightly more advocacy than evidence because they want us to say ‘there are so many deaths in the world, it’s the indicator with the biggest inequality’ etc., etc., yet the evidence base isn’t there.

Despite her discomfort, she came out of the meeting feeling ‘enlightened’, she recalled; ‘I understood that we can’t continue to make these statements, that “we can’t measure, we can’t do anything”’. Rather, she explained, she had realised that she could appeal to decision-makers by using existing data to back up more positive messages about the potential for future progress.

Similar tensions came to a head in the controversy that erupted with the publication of revised global maternal mortality estimates in 2010. For the first time, these estimates were produced not by UN statisticians and their academic counterparts, but by the newly formed Institute for Health Metrics and Evaluation in Seattle, funded by the Gates Foundation to scrutinise and revise global health data. The Institute was led by Chris Murray, who designed the influential DALY methodology in the 1980s, which had been heavily contested by maternal health specialists for disfavouring women (Sundby, 1999). Statisticians at the Seattle institute assessed levels and trends in maternal mortality for 181 countries for 1980–2008. They constructed a database from multiple sources, including vital registration data (where available), censuses, surveys and verbal autopsy studies, and used ‘robust analytical methods’ to generate modelling estimates of maternal deaths and the MMR for each year between 1980 and 2008 (Hogan et al., 2010). Their analysis suggested that the global MMR had actually decreased substantially over time, by 1.3% per year since 1990, and that the annual number of maternal deaths was now much lower than previously assumed, at around 275,000 rather than 500,000. ‘Substantial, albeit varied, progress has been made towards MDG 5’, the Institute’s statisticians concluded (Hogan et al., 2010, p. 1609).

If maternal health experts had previously worried that the publication of the same figures year after year created donor fatigue, many now worried about the implication of the new assertion about a downward trend. Would it mean that the urgency of addressing maternal health would dissipate? Or create complacency about donors’ maternal health efforts? Some worried that attributing MMR reduction to donor initiatives legitimated donors’ focus on targeted approaches, rather than broader changes to address underlying social and economic determinants of health (cf. McCoy, Jensen, Kranzer, Ferrand, & Korenromp, 2013). Significantly, they questioned, as Yamin and Boulanger (2014) have recently done, what can actually be asserted with respect to the reported progress in MMRs in specific countries, given the serious methodological limitations in how the estimates were produced. What’s more, global estimates, others highlighted, mask significant internal social inequities. ‘There is a lot of interest in having a success story’, said one British epidemiologist, but the flip side of focusing on global success is neglect of the vast disparities in maternal mortality that persist both between and within countries, which cannot be captured in the global estimates. As a senior WHO official pointed out, if overall reduction ‘was achieved by zeroing maternal mortality in the top forty percent by socioeconomic status of women of reproductive age and doing nothing for women in the bottom sixty percent … that’s not particularly encouraging’.

Significantly, the publication of the new figures also brought to the fore an emerging power struggle over who holds the authority to define global data. The fact that the Gates Foundation had funded the new estimates was widely perceived as a direct assault on WHO’s constitutional mandate to produce and disseminate health statistics, and another demonstration of the Foundation’s immense power within global health (see also Boerma, Mathers, & Abou-Zahr, 2010). Saving face, the WHO revised their own global estimates, confirming the lower total and the downward trend (WHO, 2010). But many were concerned that the challenge to WHO’s statistical authority had further damaged the scientific credibility of maternal mortality data. ‘Do people really believe the information?’ asked one epidemiologist, before answering her own question: ‘Everybody knows. Everybody has concerns about maternal mortality’.

## The ‘other’ indicators

An unintended, but significant, effect of measurement for global accountability and competition has been to divert attention from basic epidemiological monitoring data and actual measurement of maternal mortality for national and sub-national problem solving. The push to monitor progress towards global goals, notably the MDGs, has, for example, imposed pressure on governments to demonstrate success in ways that, as Yamin and Boulanger (2014, p. 113) observe, has provided incentives to invest in ‘ever more sophisticated modelling and analysis based on poor data, while national health information systems have languished. This is unfortunate since many experts agree that it is well-functioning health information systems that can best mitigate the various technical and political problems with maternal mortality estimates and play a vital role in planning and monitoring public health programmes that can respond quickly to local epidemiological profiles.

Many of the leading measurement experts we interviewed reflected self-critically on such effects, which they had observed across the countries in which they conduct research. They admitted, for instance, that much of their measurement work may serve global accountability needs and even push countries to act to improve maternal health, but does too little to address the underlying problem of poor national-level capacity to measure and analyse maternal mortality. One informant commented that, ‘it is a tragic paradox that in Africa and other low-income countries there are no *real* data, despite the fact that that’s where the world’s great majority of the burden of disease lies’. Several privately suggested that the money and effort spent on refining maternal mortality measurement techniques would have been more judiciously spent on helping countries set up proper health information systems, including vital registration, census, and routine administrative data systems at national, district or facility levels. As maternal health experts have themselves argued, nineteenth century maternal mortality declines in Western Europe benefitted greatly from localised measurement and monitoring groups for developing effective and targeted initiatives (Béhague & Storeng, 2013).

Even those who had been involved in designing and modifying global survey techniques were self-critical about the drive for ever more sophisticated, frequent and large-scale surveys to produce mortality estimates. Several of our interlocutors explained that even if they could reveal the precise magnitude of maternal mortality, this would be of limited value in informing debates about *how* to improve maternal health. As a prominent American measurement specialist with more than three decades of experience explained:

I just learnt that the DHS (Demographic Health Survey) is going to spend like a zillion dollars in Pakistan to interview, what was it, 90,000 extra households. I don’t see the point of that … You go into Pakistan and spend how many million dollars and do this extra thing and … just to tell us again that women are dying … *Of course* they’re dying in Pakistan, there’s no bloody health system! If you come out of this study and you have a MMR of 750 or of 600, what are the differences in implications? None. Doesn’t tell you a thing. It’s high. We know it’s high.

This comment resonates with the view that the MMR may not actually be the most relevant indicator for assessing country-level progress and certainly not for informing country-level policy and planning. As another American maternal health epidemiologist said, ‘I think we should be looking at other kinds of indicators. Not forgetting maternal mortality, but not going all the fifty thousand yards to making that the real end…or state of the art indicator’. In effect, she was revisiting arguments first made in the 1990s that *process* indicators – quantitative indicators of the availability, access to and quality of different healthcare services – may be better suited to assess specific countries’ progress towards the realisation of maternal health, even if there are still measurement problems associated with these indicators (cf. Yamin & Boulanger, 2014). ‘I’m not sure it’s maternal mortality we ought to be measuring,’ a leading UK-based epidemiologist pondered, and suggested the field should pay more attention to ‘trends in skilled attendance, trends in C-sections amongst the poor, which I think is a very good indicator.’ Unlike the MMR, process indicators like C-section coverage can often be disaggregated according to geographic and socio-economic variables, and can thereby be used to ensure equitable implementation of services. Such indicators, therefore, arguably respond better to country-level policy-makers and health planners’ need for context-specific data. Another advantage of process indicators, experts explained, is that they are often either available from existing recording systems at health facilities or incorporated into routine health information systems, and are thus cheaper and simpler to collect than maternal mortality data (cf. Goodburn, 2002). The epidemiologist cited above claimed that the safe motherhood field could do well to advocate more strongly for monitoring of service coverage data, rather than outcome data, even at the global level, as the Child Survival movement has done.

Others returned to the concerns first expressed in the early 1990s that the focus on mortality data is too narrow. They drew attention to the ‘neglected agenda’ of maternal *morbidity* – delivery-related problems like anaemia, maternal depression, fistula and genital and uterine prolapse – estimated to be 20 times more frequent than maternal mortality (Hardee, Gay, & Blanc, 2012; WHO, 2004). A main argument for these informants was that donors and governments should consider morbidity data in priority setting, because when combined with mortality data, morbidity data help to show that pregnancy-related causes are ‘a leading contributor to the burden of disease among women’ (Koblinsky, Chowdhury, Moran, & Ronsmans, 2012, p. 124). Crucially, maternal health experts have noted that clinical interventions designed to reduce the MMR by targeting severe obstetric emergencies, the direct causes of maternal mortality, may have little if any impact on acute or chronic morbidity, which may require other treatment options, including surgical repair of fistulas and medical management of anaemia. Judging the success of maternal health efforts in terms of the MMR alone therefore leaves open the possibility of ‘meeting the target’ of MMR reduction, but ‘missing the goal’ of improving maternal *health*.

While many prominent measurement experts have thus, in various ways, tried to push for a more diverse evidence-base that includes an array of indicators, an impediment to this agenda is that, as we were often told, in the present climate only ‘hard’ indicators of the number of lives saved truly influence donors or enable governments to gain visibility on the global scene. A senior British epidemiologist put it bluntly:

That’s the only way you get the attention of the Bank and the big people … If you just go and say, ‘well, we changed a bit of behaviour’ they say ‘thank you’. But if you can actually say, ‘we’re hitting the Millennium Development Goal,’ then it hugely raises you up the agenda.

## Discussion

Anthropologist Susan Erikson has argued that while indicators allegedly operate in the world as credible, apolitical and authoritative (Erikson, 2015, p. 1157), they are rarely neutral and are, instead, both value-laden and often, as Vincanne Adams puts it, quite ‘messy’ (Adams, 2016a, p. 59). Making numbers appear authoritative therefore requires hard work (Harper, 2006). Our findings support the observation that increasingly such work is about making ‘global’ data appear robust, requiring a high degree of “arithmetic gymnastics’ to make countries look metrically comparable (Adams, 2016b, p. 32). Indeed, in the history of maternal mortality measurement, scientific practice and measurement techniques are increasingly geared towards the data needs of donors that prioritise cost-effective logics rather than the data needs of national governments, and as such can be thought of in terms of ‘micropractices of neoliberalism’ (Adams, 2016a). Demands for evidence of the ‘number of lives saved’ as the product of return-on-investment help to explain the trend towards highly sophisticated statistical models and survey techniques to *estimate* maternal mortality at the partial expense of longer term investments national civil registration systems capable of *measuring* maternal deaths and other basic public health statistics.

At the start of the SMI, scientists were adamant that indirect estimation of maternal mortality was only an interim measure to be used until countries developed national health information systems capable of measuring maternal mortality. In reality, indirect modelling techniques have instead replaced routine information systems as the primary source of data on maternal mortality today. Experts justify research on indirect estimation techniques by claiming that such techniques produce data needed for ‘tracking progress towards the development goals and monitoring and evaluating the effectiveness of programmes,’ and to respond to ‘international concern with accountability and rational resource allocation’ (Merdad et al., 2013). But questions are rarely asked about whose accountability is at stake and whose data needs are being met.

While we have focused on the impact on global scientific practice, the effects of such global practices of course become real when they manifest in local contexts. Scholars are starting to delineate the ambivalent effects that the current obsession with numerical targets has on the health programmes and governance processes at the national and local levels, including how they breed simplification and abstraction that distort programmatic practice (Hodžić, 2013; Oni-Orisan, 2016; Wendland, 2016; Yamin & Boulanger, 2014). Claire Wendland’s ethnographic work on maternal mortality in Malawi elegantly describes how technical challenges of producing data distort and ignore the local realities of deliveries and maternal deaths, and how the numbers become mobilised as claims of success for individual political gain by Malawi’s former president and others (Wendland, 2016). Svea Closser’s (2011) study of the global Polio Eradication Initiative’s work in Pakistan analyses how officials created a culture of optimism by devaluing negative evidence. This intended and unintended manipulation of the numbers was designed to convince donors to continue funding the project, but prevented constructive analysis of the project’s problems. Similarly, in a powerful critique of the well-intentioned Norway–India Partnership intended to accelerate progress on maternal and child survival, Sidsel Roalkvam and Desmond McNeill (2016) ask whether global initiatives to finance maternal and child health change the direction of accountability, with national governments becoming accountable upwards to donors for achieving specified numerical targets, rather than downwards to their citizens. They suggest that the blending within global public health of neoliberal ideology and a shared moral purpose of maximising the number of lives saved is deeply depoliticising, distracting attention from the configurations of power that shape the policy space of nation states (Roalkvam & McNeill, 2016, p. 69).

Measurement experts are not blind to such unintended effects; in fact, they generate considerable unease and debate within the measurement community. For example, while experts recognise that global estimates can persuade policy-makers to act, they can also undermine trust. WHO statisticians Carla AbouZahr and Ties Boerma (2005, p. 581) have argued, for instance, that when global agencies and academics use modelling to fill in missing data elements, ‘countries perceive this as an externally driven process, designed to meet donor needs and of little relevance to country action’. According to Sakiko Fukuda-Parr and colleagues (2014, p. 10), ‘indicators which depend upon global estimation exercises are poorly adapted to fostering national ownership and participation of the people who are the most affected by development programming’. At the heart of the dispute is a deep-seated concern that debates about whether global maternal mortality has improved or not can be a distraction from more important debates about the maternal health situation in particular countries, and crucially, how to improve it.

The fact that such debates rarely surface in public spheres reflects that it is difficult to challenge donors’ demands for ‘global’ data, especially because they are rooted in a business-oriented ethos that co-exists with stated commitment to public health values like the imperative to save lives. While donors have undoubtedly skewed measurement work away from national needs, they also support some major initiatives to improve national health information systems.

An example can be seen in the 50 million dollar grant the Gates Foundations provided in 2005 to form the Health Metrics Network in Geneva, a global health initiative that, until its closure in 2013, aimed to ‘provide coherent and coordinated reform and strengthening of country health information systems’ (AbouZahr & Boerma, 2005, p. 583). Through health information assessments and support to more than 70 countries, it intended to redress the lack of an overall vision of a comprehensive health information system that had hampered previous international efforts. Despite such good intentions, critics like Susan Erikson (2012, p. 375) have denounced the network as a ‘Gates-by-proxy institute’ whose claims to have strengthened national health systems in countries, like Sierra Leone, are ‘a myth’. An enduring paradox is that the very donors who committed to joint funding and development of national health information systems through initiatives like the Health Metrics Network also encourage bureaucratic expansion at the country level. For instance, they impose heavy reporting requirements (Taylor & Harper, 2014) and support and implement their own data collection platforms and independent bodies for global monitoring (Storeng, 2014). Donors often support such initiatives due to concerns about the quality of data individual countries report, but doing so also paradoxically undermines national health information system strengthening.

## Conclusion

In a commentary on academics’ role in the production of health indicators, Christopher Murray and Alan Lopez (2010, p. 210) – themselves world-renowned statisticians – write about a maternal health advocate who called for scientists to be ‘locked in a room until they agree on one set of numbers’. This anecdote resonates with our informants’ stories about the pressure they felt from the broader safe motherhood community to support a coherent story about global mortality decline. Murray and Lopez go on to argue that such ‘artificial consensus’ about health statistics is fundamentally misguided because, they claim, policy-makers are sophisticated enough to understand that data on levels and trends are to some extent uncertain and are only to be used as rough guides. But their call for academics to exercise their responsibility to be open about statistical uncertainty contradicts many academics’ own experience that policy-makers either ‘don’t understand confidence intervals,’ or use uncertainty as an excuse to put their money elsewhere.

It also discounts just how deeply enmeshed academics have become within the prevailing power structures of global health governance. Maternal health research is to a large extent funded by global donors, including the Gates Foundation, which is well known for not welcoming open critique (Harman, 2016), and academic livelihoods are dependent on sustained donor interest in maternal health. As we have shown elsewhere, those who continue to conduct research using intensive, historical, case-study methods that make effective use of trend data – once a mainstay of epidemiological research – now consider this an agenda that they must do ‘on the side’, almost as a form of indirect subversion of global mortality-based demands (Béhague & Storeng, 2013). Academic researchers may feel unease about their limited responsiveness to national data needs, but as David McCoy and others have argued, they work within an academic culture in which they lack the inclination, incentives and sometimes skills to conduct the sort of research that developing countries need (McCoy, Mwansambo, Costello, & Khan, 2008, p. 1056). This partly explains why, 30 years after the first global maternal mortality estimates were produced, most MMRs continue to be based on a combination of data sources from surveys and mathematical modelling, while empirical measurement of actual maternal deaths remains scarce and measurement experts continue to question whether anybody trusts the data.

## Funding

This article is based on a collaborative research project (Béhague and Storeng) funded by the Economic and Social Research Council [grant number RES- 000–22–1039] and Storeng’s Ph.D. research, funded by the Research Council of Norway [grant number 175321], with support from the LSHTM. Storeng was funded by a Research Council of Norway Young Scientist Grant [grant number 220608] during the writing of the manuscript. The funders were not involved in determining the study design, the collection, analysis, and interpretation of data, or in writing this article.

## Disclosure statement

No potential conflict of interest was reported by the authors.
